# UBQLN4 promotes the proliferation and invasion of non-small cell lung cancer cell by regulating PI3K/AKT pathway

**DOI:** 10.1007/s00432-024-05862-8

**Published:** 2024-07-06

**Authors:** Li He, Heng Chen, Bin Ruan, Li He, Ming Luo, Yulun Fu, Rui Zou

**Affiliations:** 1 Department of Oncology, The People’s Hospital of Xinyu City, Xinyu, Jiangxi 338099 People’s Republic of China; 2 Department of Oncology, The First Affiliated Hospital, Jiangxi Medical Collge, Nanchang University, 17 Yongwai Street, Nanchang, Jiangxi 330006 People’s Republic of China; 3 Department of Pathology, Jingdezhen First People’s Hospital, Jingdezhen, Jiangxi 333000 People’s Republic of China

**Keywords:** Ubiquilin-4 (UBQLN4), Non–small cell lung cancer (NSCLC), Proliferation, Invasion, PI3K/AKT pathway

## Abstract

**Background:**

Ubiquilin-4 (UBQLN4), a member of the ubiquilin family, has received limited attention in cancer research to date. Here, we investigated for the first time the functional role and mechanism of UBQLN4 in non-small cell lung cancer (NSCLC).

**Methods:**

The Cancer Genome Atlas (TCGA) database was employed to validate UBQLN4 as a differentially expressed gene. Expression differences of UBQLN4 in NSCLC cells and tissues were assessed using immunohistochemistry (IHC) experiment and western blotting (WB) experiment. Kaplan-Meier analysis was conducted to examine the association between UBQLN4 expression and NSCLC prognosis. Functional analyses of UBQLN4 were performed through cell counting kit-8 (CCK-8), colony formation, and transwell invasion assays. The impact of UBQLN4 on tumor-associated signaling pathways was assessed using the path scan intracellular signaling array. In vivo tumorigenesis experiments were conducted to further investigate the influence of UBQLN4 on tumor formation.

**Results:**

UBQLN4 exhibited up-regulation in both NSCLC tissues and cells. Additionally, over-expression of UBQLN4 was associated with an unfavorable prognosis in NSCLC patients. Functional loss analyses demonstrated that inhibiting UBQLN4 could suppress the proliferation and invasion of NSCLC cells in both in vitro and in vivo settings. Conversely, functional gain experiments yielded opposite results. Path scan intracellular signaling array results suggested that the role of UBQLN4 is associated with the PI3K/AKT pathway, a correlation substantiated by in vitro and in vivo tumorigenesis experiments.

**Conclusion:**

We validated that UBQLN4 promotes proliferation and invasion of NSCLC cells by activating the PI3K/AKT pathway, thereby facilitating the progression of NSCLC. These findings underscore the potential of targeting UBQLN4 as a therapeutic strategy for NSCLC.

## Introduction

Lung cancer is a prevalent malignant tumor posing a significant threat to human life. It ranks second in global cancer incidence, trailing only behind breast cancer in women, and holds the top position in global cancer-related mortality, serving as a primary cause of cancer deaths worldwide(Mederos et al. 2020; Xie et al. 2021). Based on histopathological characteristics, lung cancer is primarily categorized into non-small cell lung cancer (NSCLC) and small cell lung cancer. Notably, NSCLC constitutes over 80% of all lung cancer cases(Liang et al. 2020; Xie et al. 2021). NSCLC encompasses lung squamous cell carcinoma (LUSC) and lung adenocarcinoma (LUAD)(Fan and Schraeder 2011). Despite significant progress and improvements in treatment modalities, the survival rates for NSCLC has seen some enhancement, however, due to their rapid proliferation and metastasis, long-term prognosis for NSCLC patients remains unfavorable(Guo et al. 2022; Tang et al. 2022). Therefore, unraveling the molecular mechanisms underlying the progression of NSCLC is crucial to advancing therapeutic interventions.

The ubiquitin itself is a small protein molecule, and acts as a molecular tag, directly attaches to target proteins, marking them for degradation(Bonifacino and Weissman 1998).  Ubiquilin proteins (UBQLN), members of the ubiquitin-like and ubiquitin-associated domain protein family, are crucial factors for maintaining cellular homeostasis, comprising five closely related members: UBQLN1, UBQLN2, UBQLN3, UBQLN4, and UBQLN5(Jantrapirom et al. 2020; Yu et al. 2020). UBQLN acts as an adapter protein, aiding in the targeting of ubiquitinated proteins for degradation within the proteasome(Bonifacino and Weissman 1998). UBQLN plays a role in regulating the ubiquitination pathway of protein degradation in cells and participating incellular processes such as the cell cycle, apoptosis, membrane receptors, DNA repair, epithelial-mesenchymal transition (EMT), and microRNA activity(Bonifacino and Weissman 1998; Wang et al. 2022).

UBQLN4 belongs to the UBQLN, participating in various cellular processes and often exhibiting over-expression in many tumor cells. This over-expression is associated with poor prognosis in various cancers. For instance, UBQLN4 over-expression promotes metastasis and poorly differentiated outcomes in cervical cancer, leading to adverse prognosis(Wang et al. 2022); additionally, UBQLN4 activates the Wnt-β-catenin pathway, facilitating the progression of hepatocellular carcinoma (HCC)(Yu et al. 2020); activated by C/Ebpβ, UBQLN4 promotes proliferation, migration, and invasion in colorectal cancer through the Wnt/β-catenin signaling pathway (Tang et al. 2021). A recent study indicates that UBQLN1 and UBQLN2 regulate LUAD cell survival, proliferation, migration, and the cell cycle by modulating myosin expression(Shah and Beverly 2023). However, the role of UBQLN4 in the occurrence and development of NSCLC remains unclear.

In this study, we investigated the role and regulation of UBQLN4 in NSCLC. Our findings reveal that UBQLN4 promotes the proliferation and invasion of NSCLC cells by activating the PI3K/AKT pathway, thereby facilitating tumor development.

## Materials and methods

### Analysis of UBQLN4 expression

Utilize the TCGA database to investigate the expression profile of UBQLN4 across various tumors. Compare the gene expression between cancerous tissues and adjacent non-cancerous tissues.

### Patients and samples

Tissue samples and adjacent normal tissue samples were collected from 80 NSCLC patients who were first diagnosed with NSCLC without distant metastasis in Xinyu People’s Hospital. The Ethics Committee of Xinyu People’s Hospital approved the study.

### Cell culture

Obtain NSCLC cell lines (H520, H1299, A549, H358) and human bronchial epithelial (HBE) cell line from the Chinese Academy of Sciences’ Cell Bank. H1299 cells and A549 cells are cultured in dulbecco’s modified eagle’s medium (DMEM) (Gibco) supplemented with 10% fetal bovine serum and 1% penicillin/streptomycin (100U/ml penicillin and 10 µg/ml streptomycin). H358 cells and H520 cells are cultured in roswell park memorial institute-1640 medium (Gibco) with 10% fetal bovine serum. HBE cells are cultured in DMEM-12 (Gibco) with 10% fetal bovine serum. All these cells are maintained in a humidified incubator at 37 °C with 5% CO2.

### Western blotting analysis and polymerase chain reactionassay

The protein expression of UBQLN4 in tumor tissues and cultured cells was analyzed using WB. Cells and tissues were lysed in radio-immunoprecipitation assay lysis buffer (Beyotime, Beijing, China) containing a proteinase inhibitor (Genebase, Shanghai, China), and the protein concentration was determined using the Pierce BCA Protein Assay Kit (Thermo Scientific, Waltham, MA, USA). The protein extracts were separated on 10%–12% sodium dodecyl sulfate polyacrylamide gel electrophoresis, transferred to polyvinylidene fluoride membranes (Millipore, Bedford, MA, USA), and then nonspecific binded in 5% non-fat dry milk in phosphate buffer saline (PBS) containing 0.05% Tween-20 at room temperature for 1 h. The membrane was then incubated overnight at 4 °C with specific primary antibodies, followed by incubation with secondary antibodies at room temperature for 1 h (protected from light). Immunostaining was detected using the ECL Western Blotting Detection System (Amersham, Piscataway, NJ, USA), with β-actin serving as the internal reference. Antibodies against UBQLN4 (1:1000 dilution, Santa Cruz, sc-136145), β-actin (1:1000 dilution, CST, #8480S), p-PI3K(1:1000 dilution, Abcam, #ab278545), PI3K(1:1000 dilution, Abcam, #ab32089), AKT (1:1000 dilution, CST, #9272;), P-AKT(Thr308) (1:1000 dilution, CST, #13038), E-cadherin(1:500 dilution, abcam, ab15148), Vimentin(1:500 dilution, abcam, ab137321), and N-cadherin monoclonal(1:500 dilution, abcam, ab76057) antibodies were used for sample probing.

Total ribonucleic acid (RNA) was isolated from cultured cells using Trizol (Invitrogen, USA) according to the manufacturer’s instructions. The RNA concentration and A260/A280 ratio were determined using Nano Drop 2000. Reverse transcription was performed using PrimeScript RT reagent (TAKARA, RR036A). Quantitative reverse transcription polymerase chain reaction (qRT-PCR) was conducted using Hieff^®^ qPCR SYBR Green Master Mix (Low Rox Plus) reagent produced by Yeasen Biotechnology (Shanghai) Co., Ltd.

### Immunohistochemistry

Tissue microarray chip was used to analyze 80 paired NSCLC and corresponding non-tumor tissues. It was fixed with paraformaldehyde before embedding in paraffin. After deparaffinization, the cells were incubated with anti-UBQLN4 antibody and kept in a 4 °C refrigerator overnight. After washing three times with PBS, antibody binding was detected using an HRP-DAB kit (MaxvisionTM2 HRP-Polymer antiRabbit IHC kit), and nuclei were counterstained with hematoxylin solution. Images were acquired using an Olympus microscope. IHC staining was divided into positive and negetive according to staining intensity.

### Generation of stable cell lines

The UBQLN4 overexpression plasmid (plvx-UBQLN4) and the empty vector (plvx) were obtained from Synbio Technology Co., Ltd. in Suzhou, China. The co-transfection of the empty vector plvx or plvx-UBQLN4 with packaging vectors psPAX2 and pMD2 was performed in 293T cells to generate lentivirus. Upon reaching the logarithmic growth phase, A549 and H1299 cells were separately transfected with lentiviruses carrying UBQLN4 shRNA, empty vector shRNA-NC, plvx -UBQLN4, and empty vector plvx. Stably knocked down and overexpressed cell lines were then selected using puromycin (5 µg/ml, Sigma).

### Cell counting kit-8 assay and colony formation assay assay

CCK-8 assay: Log-phase cells from each group were seeded in a 96-well culture plate at a density of 1000 cells per well with 100 µl of medium. Five parallel wells were set up for each cell type, and the absorbance at 450 nm was measured at 24 h, 48 h, 72 h, and 96 h.

Colony formation assay: Log-phase cells from each group were trypsinized to form single-cell suspensions using 0.25% trypsin solution. Cell count was performed using a cell counting chamber under an inverted microscope. Cells were then seeded in 6-well plates at a density of 1000 cells per well, with each well repeated three times, and cultured for 2 weeks. After washing twice with PBS, cells were fixed in 4% paraformaldehyde for 10 min, stained with 0.1% crystal violet for 10 min, rinsed, and air-dried. Colony formation was observed and photographed using Image J software (v1.52, National Institutes of Health, USA), and the relative colony numbers.

### Cell invasion assay

Cells were suspended in 400 µl serum-free DMEM and seeded in the upper chamber at a density of 5×10^4 cells per well. The lower chamber was filled with 800 µl of DMEM containing 10% fetal bovine serum as a chemoattractant. After 24 h of incubation at 37 °C, the cells in the upper chamber were removed with a cotton swab. The cells that invaded the membrane on the bottom of the chamber were fixed with 4% paraformaldehyde and stained with 0.5% crystal violet for 30 min. Images were captured at 100x magnification under an inverted microscope, and quantification was performed.

### Path scan intracellular signaling array

To examine the activation of UBQLN4-related signaling pathways in cells, we used the path scan intracellular signaling array. Intracellular signals were detected using the primary PathScan^®^ Intracellular Signaling Array Kit #7744(Cell Signaling Technologies) following the manufacturer’s instructions. Image Studio Ver3.1 software was used for quantitative analysis of the data, and further WB was used to detect the results.

### In vivo tumorigenesis assay

This animal experiment was approved by the Animal Management Committee of the Xinyu People’s Hospital. All experimental procedures and animal care adhered to the institutional ethical guidelines for animal-related experiments.

Nude mice aged 4 to 6 weeks were obtained from (the National Laboratory Animal Center (Beijing, China)), bred under specific pathogen-free conditions, and provided with distilled water for consumption. Under sterile conditions, 2×10^6 A549/H1299 cells in 120 µl PBS were injected into the inner thigh muscles of the left (plvx -NC/sh-NC) and right (plvx-UBQLN4/sh-UBQLN4) hind legs of each mouse, with three mice per group. The mice were observed daily for general conditions.

Tumor size was measured every 7 days using calipers, and the long diameter (a, in mm) and short diameter (b, in mm) of the tumors on the mice’s body surface were measured. Tumor volume was calculated using the formula V = ab^2/2. A tumor growth curve was generated based on the tumor volume measured every 7 days. On day 28 after cell injection, mice bearing tumors were euthanized, and the tumors with intact histology were excised, weighed, and photographed.

### Statistical analysis

Statistical analysis and graphical representation were conducted using Statistical Product and Service Solutions 27.0, Adobe Illustrator 28.5.0.123 and GraphPad Prism 7 software. Independent sample t-tests or one-way analysis of variance were employed to compare mean differences between two or multiple groups, respectively. P-value< 0.05 was considered statistically significant.

## Results

### UBQLN4 is highly expressed in NSCLC and correlated with poor prognosis

**Fig. 1:**
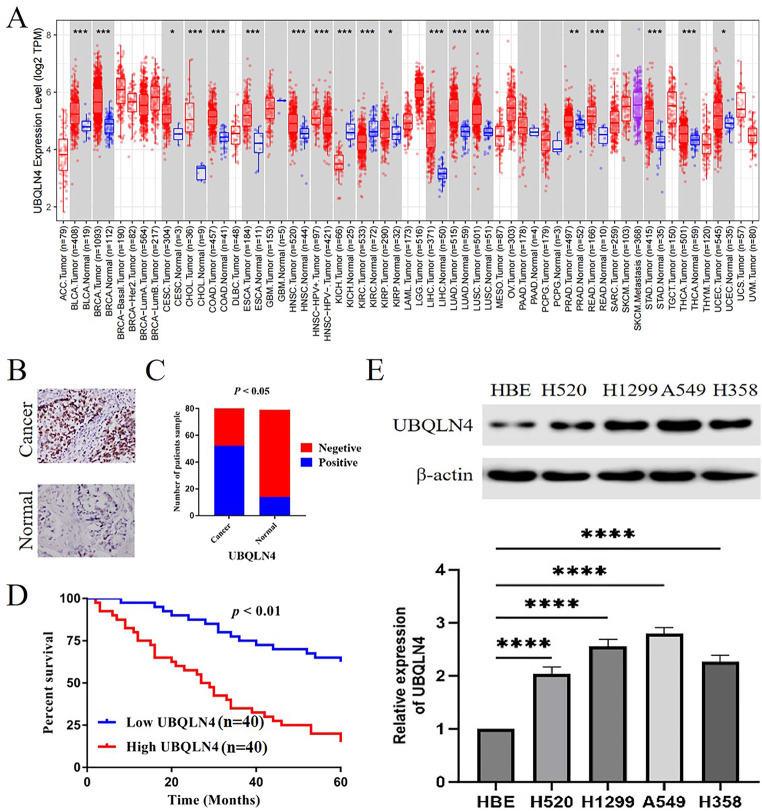
Expression level of UBQLN4 in pan-cancer and the prognostic value of UBQLN4 expression level in NSCLC patients. **A** Expression levels of UBQLN4 analyzed in TCGA database. **B** IHC was used to detect the expression of UBQLN4 in 80 cases of NSCLC and corresponding non-tumor tissues. **C** Expression of UBQLN4 in tumor tissues and adjacent normal tissues of 80 patients with NSCLC. **D** Kaplan-Meier analysis of the correlation between UBQLN4 expression and prognosis in NSCLC patients. **E** WB analysis of UBQLN4 expression in four NSCLC cell lines (H520, H1299, A549, H358) and the normal bronchial/tracheal epithelial cell line HBE. *p< 0.05, **p< 0.01, ***p< 0.001,****p<0.0001

Firstly, we conducted a pan-cancer analysis of UBQLN4 using the TCGA database. The distribution of UBQLN4 gene expression levels, as calculated through wilcoxon tests, is depicted in the box plot shown in Fig. 1A. We observed upregulation of UBQLN4 in various cancers compared to normal tissues, including BLCA, BRCA, CESC, CHOL, LUAD, LUSC, among others. In NSCLC, the increased expression of UBQLN4 may play a crucial role in the occurrence and development of the disease. For further verification, we evaluated the expression level of UBQLN4 in 80 NSCLC patients’ tissues and adjacent normal tissues using IHC, and the results showed that UBQLN4 was low in normal lung tissues but high in lung cancer tissues(Fig. 1B,Fig. 1C), *p*< 0.05. In addition, according to Kaplan-Meier survival curve analysis, NSCLC patients with high UBQLN4 expression had a poorer survival rate (Fig. 1D), *p*< 0.05. Subsequently, we examined the expression of UBQLN4 in four NSCLC cell lines (H520, H1299, A549, H358) and HBE cells to further investigate its role. WB results (Fig. 1E) revealed that UBQLN4 is highly expressed in NSCLC cell lines(H520, H1299, A549, H358), whereas it is lowly expressed in the normal bronchial/tracheal epithelial cell line HBE, *p*< 0.0001.

### UBQLN4 promotes the proliferation of more cells in NSCLC

To investigate the role of UBQLN4 in NSCLC, we selected the A549 and H1299 cell lines. A549 cells were silenced for UBQLN4 expression using lentiviral-mediated UBQLN4 shRNA, while H1299 cells were engineered for UBQLN4 overexpression using lentiviral-mediated plvx-UBQLN4. Subsequent validation through qRT-PCR and WB confirmed the successful construction.

**Fig. 2:**
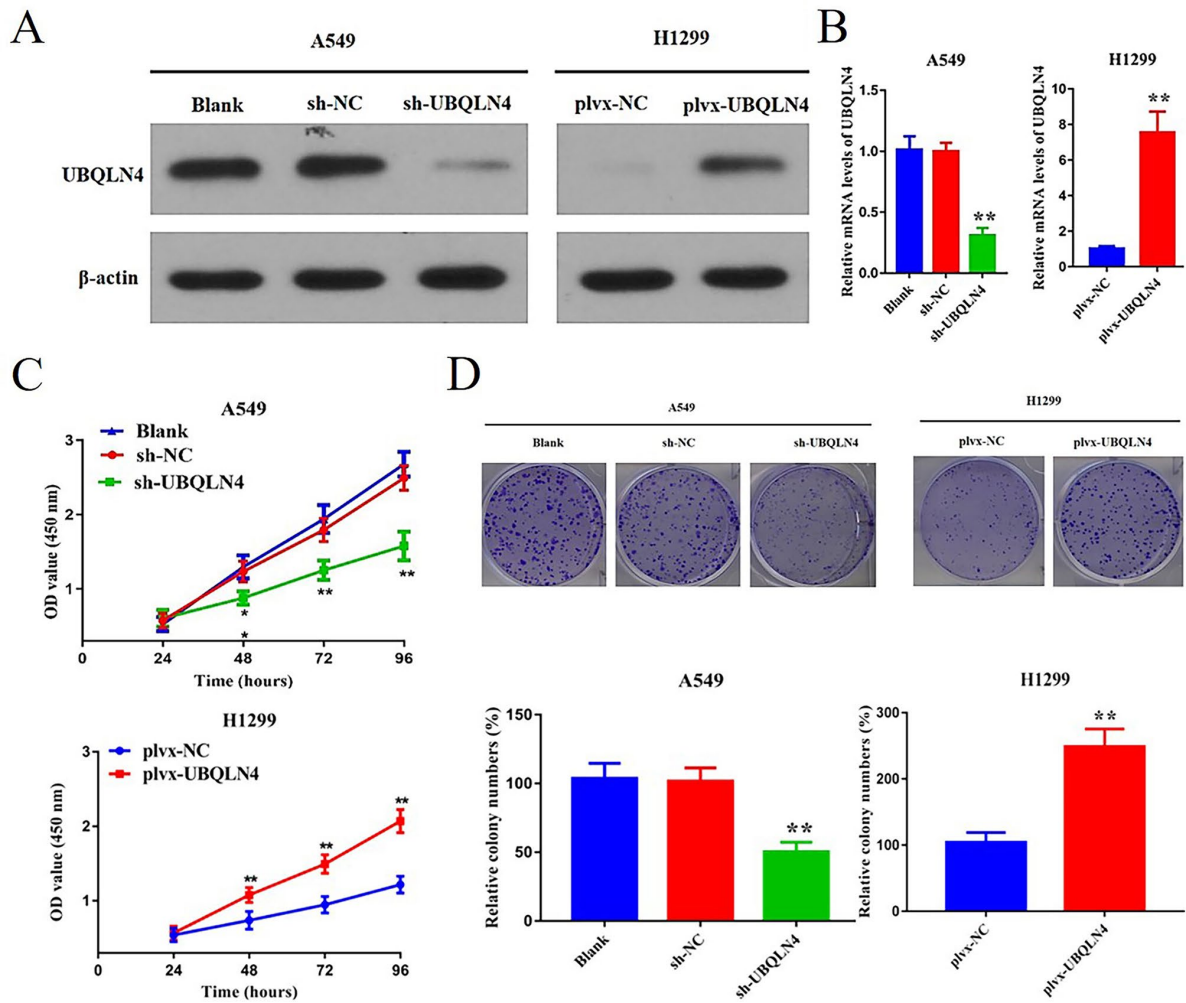
UBQLN4 promotes cell proliferation in NSCLC **A** Detection of UBQLN4 protein expression levels in knockdown and overexpression groups using WB. **B** qRT-PCR analysis of UBQLN4 mRNA expression levels in knockdown and overexpression groups. **C** CCK-8 assay was used to detect the proliferation ability of cells in UBQLN4 knockdown group or overexpression group. **D** Colony formation assay was used to detect the colony formation ability of cells in UBQLN4 knockdown group or UBQLN4 overexpression group. *p < 0.05, **p< 0.01, ***p< 0.001, ****p＜0.0001

The results demonstrated a significant reduction in UBQLN4 protein and messenger RNA (mRNA) expression in UBQLN4 shRNA-transfected cells and an elevation in UBQLN4 protein and mRNA expression in plvx-UBQLN4-transfected cells, both with *p*< 0.01 (Fig. 2A,Fig. 2B), confirming the successful establishment of UBQLN4 overexpression and knockdown cell lines.

Next, we explored the impact of UBQLN4 on cell proliferation using CCK-8 and colony formation assay. CCK-8 results revealed a significant inhibition of cell proliferation in the UBQLN4 knockdown group compared to the control (sh-NC) group (*p*< 0.01). Conversely, in the overexpression group, cell proliferation was faster in the UBQLN4 overexpression group than in the control group (plvx-NC) (Fig. 2C). Subsequent colony formation assays validated these findings, showing fewer colonies in the UBQLN4 knockdown group compared to the sh-NC group (*p*< 0.01) and more colonies in the UBQLN4 overexpression group compared to the control group (*p*< 0.01) (Fig. 2D). Results from both CCK-8 and colony formation assay collectively suggest that UBQLN4 promotes cell proliferation in NSCLC.

### UBQLN4 ehances the invasion ability of NSCLC cells by inducing EMT

**Fig. 3:**
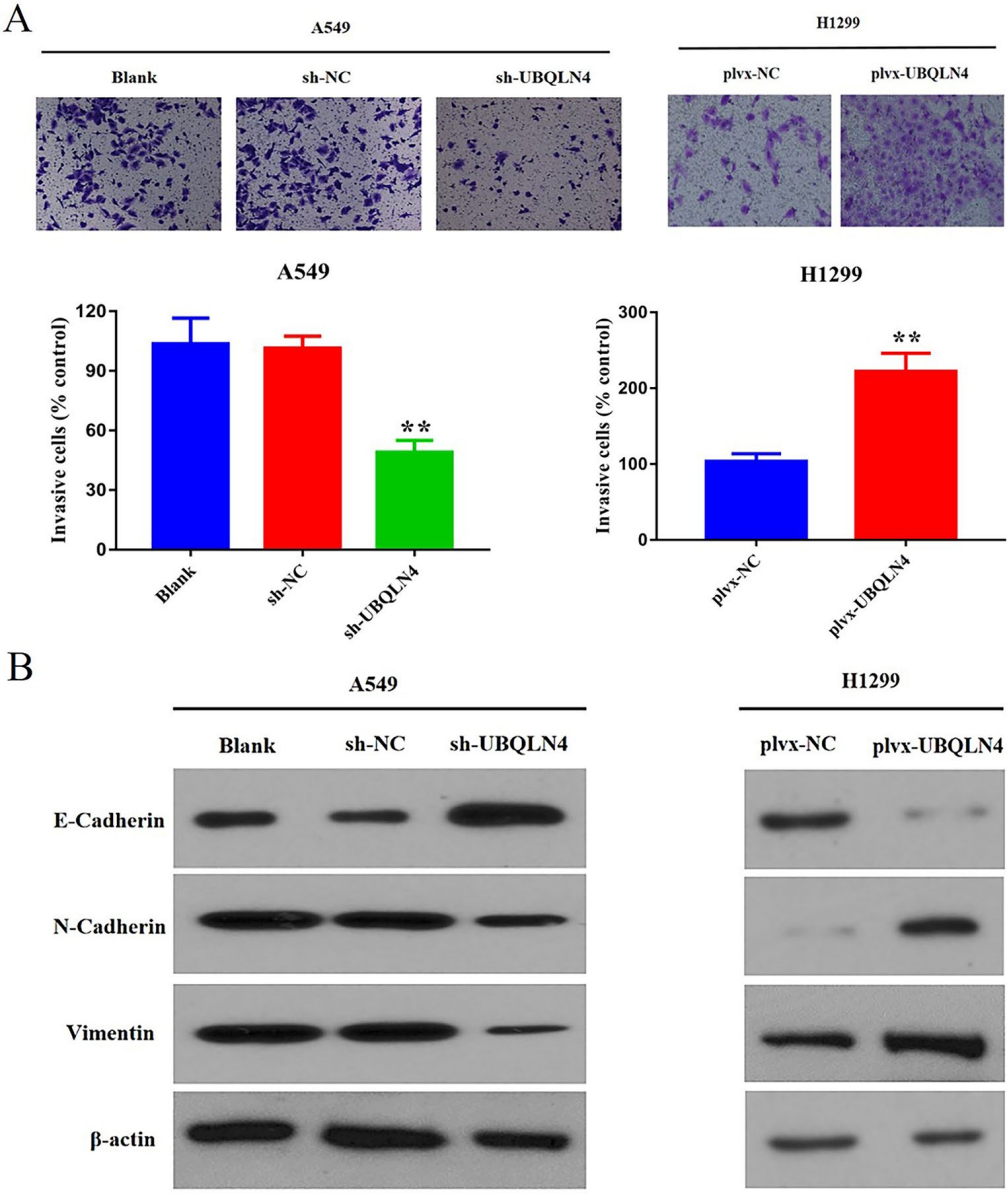
UBQLN4 enhances the invasion ability of NSCLC cells by inducing EMT **A** Cell invasion assay was used to detect the invasion ability of cells in UBQLN4 knockdown group and overexpression group. **B** WB analysis of EMT markers (E-cadherin, N-cadherin, and vimentin) expression in UBQLN4 knockdown and overexpression groups. *p< 0.05, **p< 0.01, ***p< 0.001,****p＜0.0001.

Next, we conducted transwell experiments to further investigate the impact of UBQLN4 on the invasive ability of NSCLC cells. The results revealed that UBQLN4 knockdown reduced cell invasion capacity, while conversely, UBQLN4 overexpression enhanced cell invasion capability, **p< 0.01 (Fig. 3A). EMT is a process in which epithelial cells acquire mesenchymal characteristics. Following EMT, epithelial cells lose their epithelial features and gain mesenchymal characteristics. In cancer, this process promotes tumor cell invasion and metastasis(Brabletz et al. 2021; Pastushenko and Blanpain 2019). Therefore, we utilized WB analysis to explore the connection between UBQLN4 and EMT. We found that, compared to the control group, UBQLN4 knockdown led to an increase in the expression of epithelial markers (E-cadherin) and a reduction in the expression of mesenchymal markers (N-cadherin and vimentin). Conversely, the overexpression group exhibited opposite results (Fig. 3B). Taken together, these results suggest that elevated UBQLN4 expression enhances EMT in epithelial cells, weakening cell-cell contacts and augmenting cell invasion and migration capabilities.

### UBQLN4 promotes NSCLC progression via the PI3K/AKT pathway

**Fig. 4:**
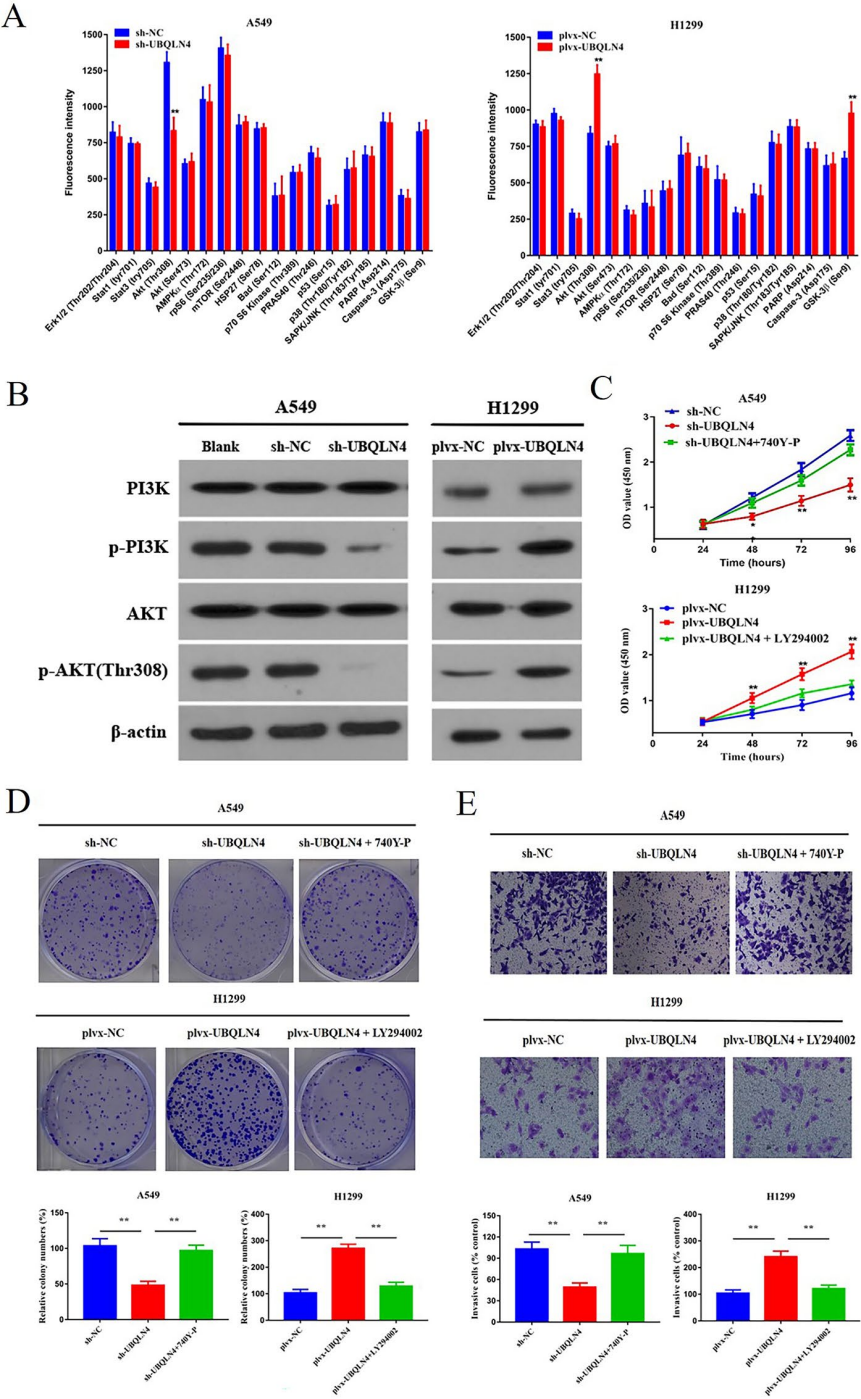
UBQLN4 promotes NSCLC cell proliferation and invasion through PI3K/AKT pathway activation **A** A549 and H1299 cells were transfected with sh-UBQLN4 and plvx-UBQLN4, respectively, and 24 h later, path scan intracellular signaling array was employed to assess signal pathways associated with UBQLN4 in NSCLC cells. **B** WB analysis was conducted to evaluate the protein expression levels of PI3K, p-PI3K, AKT, and p-AKT in UBQLN4 knockdown and overexpression groups. **C** CCK-8 assay was used to detect the proliferation ability of cells adding PI3K activator (740Y-P) and PI3K inhibitor (LY294002) UBQLN4 knockdown group and overexpression groupin UBQLN4 knockdown and overexpression groups. **D** Colony formation assay was used to detect the colony formation ability of cells adding PI3K activator (740Y-P) and PI3K inhibitor (LY294002) UBQLN4 knockdown group and overexpression group. **E** transwell assay was used to detect the invasion ability of cells adding PI3K activator (740Y-P) and PI3K inhibitor (LY294002) UBQLN4 knockdown group and overexpression group. *p＜0.05,, **p< 0.01, ***p< 0.001,****p＜0.0001.

To further investigate how UBQLN4 promotes the proliferation and invasion of NSCLC cells, we utilized the PathScan^®^ intracellular signaling array to screen for signal pathways associated with UBQLN4 in NSCLC cells. Cell signaling in A549 cells before and after UBQLN4 knockdown and in H1299 cells before and after UBQLN4 overexpression were compared. As shown in Fig. 4A, compared with the control group, the phosphorylation of AKT (Thr308) was down-regulated in sh-UBQLN4 group, while the phosphorylation of AKT (Thr308) and glycogen synthase kinase-3β (GSK-3β)  (Ser9) was up-regulated in plvx-UBQLN4 group. To further verify this result, we also employed WB analysis to detect changes in PI3K, p-PI3K, AKT, and p-AKT in NSCLC cells with UBQLN4 knockdown and overexpression. After UBQLN4 knockdown, the protein levels of p-PI3K and p-AKT decreased, while overexpression led to the opposite result, with increased levels of p-PI3K and p-AKT proteins (Fig. 4B). Therefore, we preliminarily conclude that UBQLN4 promotes NSCLC cell proliferation and invasion by activating the PI3K/AKT pathway to some extent. To further validate these results, we conducted additional experiments. We introduced a PI3K activator (740Y-P) into the UBQLN4 knockdown group and a highly selective PI3K inhibitor (LY294002) into the overexpression group. Subsequently, we performed CCK-8, colony formation, and cell invasion assay to explore changes in cell proliferation and invasion. The results demonstrated that in the UBQLN4 knockdown group with the addition of the PI3K activator (740Y-P), compared to the sh-UBQLN4 group, cell proliferation and invasion capabilities were enhanced. Conversely, in the overexpression group with the addition of the PI3K inhibitor (LY294002), cell proliferation and invasion capabilities were reduced, **p< 0.01 (Fig. 4C,Fig. 4D,Fig. 4E). These findings further confirm that UBQLN4 promotes NSCLC development by activating the PI3K/AKT pathway.

### UBQLN4 accelerates tumor growth in vivo

**Fig. 5:**
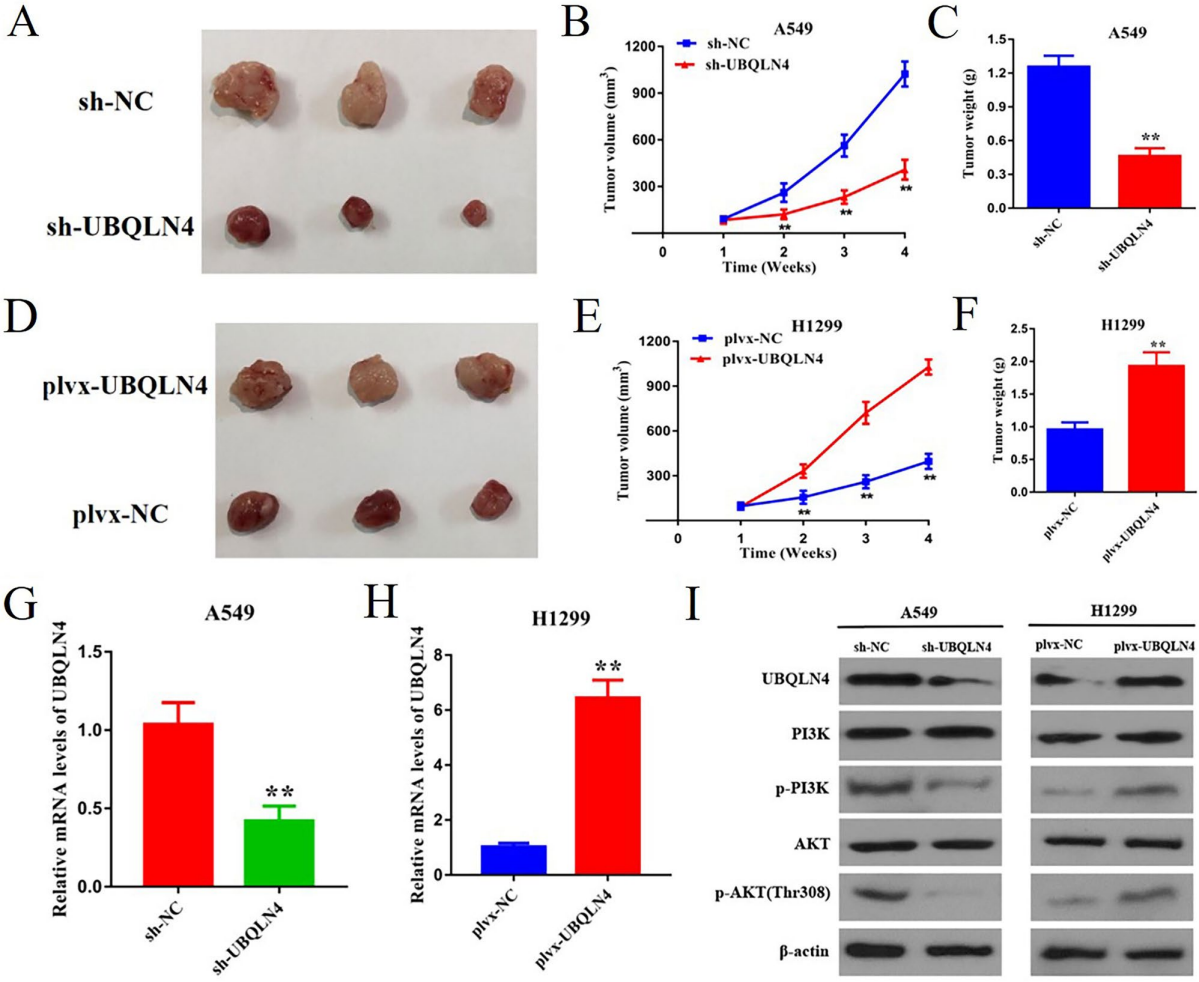
UBQLN4 promotes tumor development in vivo **A** Image of the tumor obtained on day 28 of the experiment. **B** Tumor volumes measured every 7 days in the sh-NC group and sh-UBQLN4 group (mean ± SD; *n* = 3 mice per group). **C** Tumor weights measured at the endpoint in the sh-NC group and sh-UBQLN4 group. **D** Image of the tumor obtained on day 28 of the experiment. **E** Tumor volumes measured every 7 days in the plvx-NC group and plvx-UBQLN4 group (mean ± SD; *n* = 3 mice per group). **F** Tumor weights measured at the endpoint in the plvx -NC group and plvx -UBQLN4 group. **G** Expression of UBQLN4 mRNA in tumor tissues from the shNC group, sh-UBQLN4 group. **H** Expression of UBQLN4 mRNA in tumor tissues from the plvx-NC group and plvx -UBQLN4 group. **I** Protein expression levels of UBQLN4, PI3K, p-PI3K, AKT, and p-AKT in the sh-NC group, sh-UBQLN4 group, plvx -NC group, and plvx -UBQLN4 group detected by WB analysis. * *p*< 0.05, **p < 0.01, ***p< 0.001, ****p＜0.0001.

We investigated the in vivo role of UBQLN4 using a mouse model. H1299 cells transfected with plvx-NC and plvx-UBQLN4, as well as A549 cells transfected with sh-NC and sh-UBQLN4, were implanted into the left (plvx-NC/sh-NC) and right (plvx-UBQLN4/sh-UBQLN4) inner thighs of nude mice to establish xenograft tumor models for subsequent experiments. Consistent with our previous in vitro cell experiments, we observed that the tumor volume and weight were significantly smaller in the UBQLN4 knockdown group (sh-UBQLN4) compared to the control group (sh-NC), **p < 0.01 (Fig. 5A-C). Conversely, the tumor volume and weight were significantly larger in the UBQLN4 overexpression group (plvx-UBQLN4) compared to the control group (plvx-NC), **p< 0.01 (Fig. 5D-F). These findings indicate that UBQLN4 promotes NSCLC growth.

Subsequently, we conducted qRT-PCR and WB experiments on tumor tissues obtained from mice to investigate the expression levels of UBQLN4, PI3K, p-PI3K, AKT, and p-AKT. The results revealed that in the sh-UBQLN4 group, both UBQLN4 mRNA and protein levels in tumor tissues decreased, as did the levels of p-PI3K and p-AKT proteins. Conversely, in the plvx-UBQLN4 group, the results were opposite, **p＜ 0.01 (Fig. 5G-I). In combination with the in vitro results, we conclude that UBQLN4 promotes NSCLC occurrence by activating the PI3K/AKT signaling pathway.

## Discussion

UBQLN, consisting of UBQLN1, UBQLN2, UBQLN3, UBQLN4, and UBQLN5, is a crucial factor in maintaining intracellular protein stability(Jantrapirom et al. 2020; Yu et al. 2020). Implicated in neurodegenerative diseases such as alzheimer’s disease, amyotrophic lateral sclerosis (Shah et al. 2015) and dementia(Liu et al. 2013). UBQLN plays a pivotal role in cellular processes like autophagy(N’Diaye et al. 2009)and apoptosis(Beverly et al. 2012). Emerging evidence indicates the involvement of UBQLN in human cancers. For instance, the loss of UBQLN1 is associated with enhanced cell migration and induction of EMT (Shah et al. 2015), silencing UBQLN2 activates P38MAPK, enhancing the radiosensitivity of esophageal squamous cell carcinoma(Wang et al. 2023).

UBQLN4, a member of the ubiquilin family, shares common characteristics with other UBQLN(Liu et al. 2021). UBQLN4 is associated with various cellular processes, including apoptosis(Huang et al. 2018), amyotrophic lateral sclerosis(Thakur et al. 2020), immune-related therapies(Li et al. 2021) and DNA damage repair(Jachimowicz et al. 2019). However, research on UBQLN4 in cancer is limited. Study by Huang et al.(Huang et al. 2019) demonstrated that UBQLN4 induces senescence and G1-S cell cycle arrest in gastric cancer cells, inhibiting gastric cancer growth through dual regulation of p21 in a p53-dependent and non-p53-dependent manner. An observational study by Wang et al.(Wang et al. 2022) found that cervical cancer patients with high UBQLN4 expression had lower overall survival and progression-free survival. Additionally, Yu et al.‘s research(Yu et al. 2020) revealed that UBQLN4 promotes HCC progression by activating the Wnt-β-catenin pathway. This study represents the first exploration of UBQLN4’s role in NSCLC. Our findings indicate elevated expression of UBQLN4 in NSCLC tissues, correlating with poorer overall survival. Functional experiments revealed that inhibiting UBQLN4 significantly suppressed cell proliferation, invasive capacity, and in vivo and in vitro tumorigenicity. Conversely, UBQLN4 overexpression produced opposite results. Therefore, UBQLN4 may serve as a molecular marker for prognosis and personalized therapy in NSCLC patients.

EMT, characterized by the polarization of epithelial cells convertes into mesenchymal cells, disrupts cell-cell adhesion, enhances cellular motility, and promotes the invasion and migration of tumor cells(Cai et al. 2022). TGF-β signaling pathway involves in EMT process control (Hao, Baker, & Ten Dijke, 2019). Has been reported that TGF–β was a link between EMT and PI3K/AKT(Huang et al. 2023). We investigated the association between UBQLN4 and EMT, revealing that decreases UBQLN4 expression in NSCLC cells led to an increase in the epithelial marker (E-cadherin) and a decrease in mesenchymal markers (N-cadherin and vimentin). This shift indicated a weakened EMT process in NSCLC cells with low UBQLN4 expression. Thus, the enhanced invasive capability of NSCLC cells with high UBQLN4 expression is correlated with intensified EMT. Our subsequent study found that UBQLN4 expression affected the activation of PI3K/AKT signaling pathway. We can speculate with the above data that some important genes regulating the EMT process are also affected by this pathway.

Through bioinformatic analysis, we identified that UBQLN4 expression influences the activation of the PI3K/AKT signaling pathway. PI3K, a family of intracellular lipid kinases phosphorylating phosphatidylinositol and its 30-hydroxyl derivative, play a pivotal role in cancer development. AKT, a member of the AGC (PKA/PKG/PKC) protein kinase family, consists of three homologous proteins: AKT1, AKT2, and AKT3 (Engelman et al. 2006; Murthy et al. 2000; Peng et al. 2022). The PI3K/AKT pathway is associated with drug resistance, apoptosis, proliferation, metastasis, and invasion in lung cancer(Gong et al. 2022; Guan et al. 2021; Liu et al. 2020; Xiong et al. 2022). Overactivation of the PI3K/AKT pathway promotes tumor cell proliferation, migration, and invasion(Jiang et al. 2018; Qu et al. 2021). Therefore, investigating the relationship between UBQLN4 and this signaling pathway is crucial for the treatment of NSCLC. Our study results demonstrate that UBQLN4 knockdown significantly inhibits the activation of the PI3K/AKT pathway. Additionally, reversing the effects by introducing a PI3K activator after UBQLN4 knockdown restores NSCLC cell proliferation and invasion. Activated AKT kinase is able to regulate the function of a variety of proteins involved in G1/S and G2/M progression. For example, it through its downstream substrates of GSK-3β phosphorylation C-MYC (Thr58). This process is associated with ubiquitin-dependent proteolysis(Ahmed et al. 1997; Sears et al. 2000; Xu et al. 2012). It has been found that UBQLN4 affects DNA damage repair, leading to genomic instability and promoting cancer occurrence(Jachimowicz et al. 2019). Gradually, UBQLN4 received more and more attention in the role of cancer. According to the above results, it suggests that UBQLN4 may influence the occurrence of NSCLC cells by modulating the PI3K/AKT pathway.

There have been many studies have found that MYC(Wei et al. 2019),mTOR(Wang et al. 2018; Xu et al. 2024),SOX2(Yang et al. 2023),Bcl-2(Chen et al. 2022)and so on, as downstream signaling molecules of PI3K/AKT, promote the proliferation and metastasis of NSCLC. However, path scan intracellular signaling array analysis in this study showed that the phosphorylation of these factors was not changed, while the phosphorylation of GSK-3β (ser9) was upregulated in UBQLN4 overexpression group. Kim, S. A. et al(Kim et al. 2018)investigated the anticancer effect of cryptotanshinone in NSCLC cell lines, which induced cell cycle arrest and apoptosis by Inhibited the PI3K/Akt/GSK-3β pathway. GSK-3β is an evolutionarily conserved serine/threonine kinase that plays a regulatory role in apoptosis, cell cycle, DNA repair, tumor growth, invasion and metastasis(He et al. 2020; Sahin et al. 2019). Path scan intracellular signaling array analysis showed that the phosphorylation of GSK-3β (ser9) was not changed in UBQLN4 knockdown group, while it was up-regulated in UBQLN4 overexpression group. Therefore, UBQLN4 control mechanisms in the development of NSCLC is very complex. Whether it promotes the proliferation and invasion of NSCLC cells through the PI3K/AKT/GSK-3β signaling pathway still needs to be further studied.

In conclusion, UBQLN4 regulates the proliferation and invasion of NSCLC cells through the PI3K/AKT pathway, thereby influencing the development of NSCLC. However, further studies are needed to continue exploring the downstream signaling of PI3K/AKT in the future. Perhaps in the future, UBQLN4 holds potential as a therapeutic target for NSCLC.

## Conclusion

Our data indicated that UBQLN4’s level is upregulated in NSCLC tissues, and elevated expression of UBQLN4 is associated with poor prognosis. High expression of UBQLN4 correlates with enhanced EMT, thereby promoting the invasion and migration of NSCLC cells. Furthermore, we have observed that UBQLN4 can stimulate the proliferation and invasion of NSCLC cells by activating the PI3K/AKT pathway, contributing to the progression of NSCLC. Therefore, UBQLN4 holds promise as a novel biomarker and therapeutic target for NSCLC.

## Data Availability

No datasets were generated or analysed during the current study.

## Abbreviations

UBQLN4: Ubiquilin-4
NSCLC: Non small cell lung cancer
LUSC: Lung squamous cell carcinoma
LUAD: Lung adenocarcinoma
UBQLN: Ubiquilin protein
TCGA: The Cancer Genome Atlas
HCC: Hepatocellular carcinoma
IHC: Immunohistochemistry
WB: Western blotting
CCK-8: Cell counting kit-8
DMEM: Dulbecco’s modified eagle’s medium
RNA: Ribonucleic acid
PBS: Phosphate buffer saline
mRNA: Messenger RNA
EMT: Epithelial-mesenchymal transition
qRT-PCR: Quantitative reverse transcription polymerase chain reaction
GSK-3β: Glycogen synthase kinase-3β
